# Use of Biologic or Targeted Synthetic Disease-Modifying Antirheumatic Drugs and Cancer Risk

**DOI:** 10.1001/jamanetworkopen.2024.46336

**Published:** 2024-11-20

**Authors:** Xavier Sendaydiego, Laura S. Gold, Maureen Dubreuil, James S. Andrews, Pankti Reid, David F. L. Liew, Radjiv Goulabchand, Abha Goyal Singh, Grant C. Hughes, Mathilde Pioro, Jeffrey A. Sparks, Jeffrey G. Jarvik, Siddharth Singh, Jean W. Liew, Namrata Singh

**Affiliations:** 1 Internal Medicine, University of Washington, Seattle; 2 Department of Radiology, University of Washington, Seattle; 3 University of Washington Clinical Learning, Evidence, and Research Center for Musculoskeletal Disorders, Seattle; 4 Boston University Chobanian & Avedisian School of Medicine, Boston, Massachusetts; 5 Division of Rheumatology, Veterans Affairs Boston Healthcare System, Boston, Massachusetts; 6 University of Alabama at Birmingham; 7 Veterans Affairs Birmingham/Atlanta Geriatric Research Education and Clinical Center, Birmingham, Alabama; 8 Section of Rheumatology, Department of Medicine, University of Chicago Medical Center, Chicago, Illinois; 9 Division of Rheumatology, Austin Health, Melbourne, Victoria, Australia; 10 University of Melbourne, Melbourne, Victoria, Australia; 11 IDESP, University of Montpellier, INSERM, Department of Internal Medicine, Nîmes University Hospital, Nîmes, France; 12 Division of Gastroenterology, University of California, San Diego; 13 Division of Rheumatology, Department of Internal Medicine, University of Washington, Seattle; 14 Brigham and Women’s Hospital and Harvard Medical School, Boston, Massachusetts

## Abstract

**Importance:**

The Oral Rheumatoid Arthritis Trial Surveillance demonstrated an increased cancer risk among patients with rheumatoid arthritis (RA) taking tofacitinib compared with those taking tumor necrosis factor inhibitors (TNFis). Although international cohort studies have compared cancer outcomes between TNFis, non-TNFi drugs, and Janus kinase inhibitor (JAKis), their generalizability to US patients with RA is limited.

**Objective:**

To assess the comparative safety of TNFis, non-TNFi drugs, and JAKis among US patients with RA (ie, the cancer risk associated with the use of these drugs among these patients).

**Design, Setting, and Participants:**

This retrospective cohort study used US administrative claims data from Merative Marketscan Research Databases from November 1, 2012, to December 31, 2021. Follow-up occurred up to 2 years after initiation of biologic or targeted synthetic disease-modifying antirheumatic drugs (DMARDs). Participants included individuals aged 18 to 64 years with RA, identified using at least 2 RA *International Classification of Diseases, Ninth Revision* or *International Statistical Classification of Diseases and Related Health Problems, Tenth Revision* diagnostic codes on or before the date of TNFi, non-TNFi, or JAKi initiation (“index date”). Statistical analysis took place from June 2022 to September 2024.

**Exposures:**

New initiations of TNFis, abatacept, interleukin 6 inhibitors (IL-6is), rituximab, or JAKis. Individuals could contribute person-time to more than 1 treatment exposure if treatment escalation mimicked typical clinical practice but were censored if they switched to a previously trialed medication class.

**Main Outcomes and Measures:**

Incident cancer, excluding nonmelanoma skin cancer, after at least 90 days and within 2 years of initiation of biologic or targeted synthetic DMARDs. Outcomes were associated with the most recent drug exposure.

**Results:**

Of the 25 305 individuals who initiated treatment and who met the inclusion criteria, most were female (19 869 [79%]), had a median age of 50 years (IQR, 42-56 years), and were from the South US (12 516 [49%]). Of a total 27 661 drug exposures, drug initiations consisted of 20 586 TNFi exposures (74%), 2570 JAKi exposures (9%), 2255 abatacept exposures (8%), 1182 rituximab exposures (4%), and 1068 IL-6i exposures (4%). Multivariable Cox proportional hazards regression analysis showed that rituximab was associated with a higher risk of incident cancer compared with TNFis (hazard ratio [HR], 1.91; 95% CI, 1.17-3.14), followed by abatacept (HR, 1.47; 95% CI, 1.03-2.11), and JAKis (HR, 1.36; 95% CI, 0.94-1.96).

**Conclusions and Relevance:**

In this cohort study of individuals with RA and new biologic or targeted synthetic DMARD exposures, individuals initiating rituximab, abatacept, and JAKis demonstrated higher incidence rates and statistically significantly increased risks of incident cancers compared with those initiating TNFis in the first 2 years after initiation of biologic or targeted synthetic DMARDs. Given the limitations of administrative claims data and confounding by indication, it is likely that these patients may have a higher disease burden, resulting in channeling bias. To better understand these associations, larger studies with longer follow-up time are needed.

## Key Points

**Question:**

How does the risk of incident cancer differ with the use of tumor necrosis factor inhibitors (TNFis), non-TNFis, or Janus kinase inhibitors (JAKis) in a US population of patients with rheumatoid arthritis?

**Findings:**

In this cohort study consisting of 25 305 individuals who initiated treatment with biologic or targeted synthetic disease-modifying antirheumatic drugs, a statistically significantly higher risk of incident cancer was seen with rituximab, abatacept, and JAKis compared with TNFis. Individuals initiating rituximab likely had a higher risk of incident cancer due to channeling bias, rather than the drug itself increasing cancer risk.

**Meaning:**

Larger studies with longer follow-up time are needed with careful attention to minimize channeling bias.

## Teaser

This cohort study assesses the association of tumor necrosis factor inhibitors, non–tumor necrosis factor inhibitor drugs, and Janus kinase inhibitors with incident cancer risk among patients with rheumatoid arthritis.

## Introduction

In keeping with current guidelines, treatment of rheumatoid arthritis (RA) that is refractory to conventional synthetic disease-modifying antirheumatic drugs (DMARDs) involves the use of biologic or targeted small molecular synthetic DMARDs, such as tumor necrosis factor inhibitors (TNFis), non–tumor necrosis factor inhibitors (non-TNFis: rituximab, interleukin 6 inhibitors [IL-6is], and abatacept), and Janus kinase inhibitors (JAKis).^1,2,3,4,5^ However, the recent postmarketing Oral Rheumatoid Arthritis Trial (ORAL) Surveillance demonstrated increased incidence and risk of cancer with tofacitinib compared with TNFis.^6^ Although that trial was performed among people older than 50 years with at least another risk factor for cardiovascular disease, it has raised concerns over the safety of continued JAKi use in patients with RA.

These findings necessitate closer inspection of the safety of approved biologic or targeted synthetic DMARD therapies for RA, particularly through observational studies and meta-analyses of clinical data that also encompass populations not studied in ORAL Surveillance. Khosrow-Kavar et al^7^ compared the risk of incident cancer (excluding nonmelanoma skin cancer [NMSC]) with tofacitinib vs TNFis using both a US clinical treatment cohort and a target trial emulation study that replicated the inclusion and exclusion criteria of ORAL Surveillance. In both cohorts, there was no statistically significant increased risk of cancer with tofacitinib compared with TNFis. Similarly, Huss et al^8^ performed a cohort study using Swedish registry data comparing the risk of cancer development (including NMSC) in patients with RA who had initiated treatment with a JAKi, TNFi, or non-TNFi. Although there was no increased risk of most cancers for all drug classes, there was a statistically significantly increased risk of NMSC in patients taking JAKis.

Although these studies furthered our knowledge of the comparative safety profiles of biologic or targeted synthetic DMARD therapies, their generalizability in US patients with RA is limited. Although the analysis of Khosrow-Kavar et al^7^ involved US clinical treatment cohorts, they compared outcomes only between JAKis and TNFis without analyzing other non-TNFi exposures. Huss et al^8^ compared outcomes among the 3 biologic or targeted synthetic DMARD groups, but the external validity of their findings for US patients is questionable given the demographic differences between populations in Sweden and the US. Thus, our objective was to assess the comparative safety of TNFis, non-TNFi drugs, and JAKis in patients with RA for the risk of incident cancer using US administrative claims data.

## Methods

### Data Source

We performed a cohort study using the Merative Marketscan Research Databases, which contain inpatient, outpatient, and pharmacy claims data for approximately 273 million individuals covered by employer-sponsored commercial health insurance throughout all US states and territories.^9^ They include information such as *International Classification of Diseases, Ninth Revision* (*ICD-9*) or *International Statistical Classification of Diseases and Related Health Problems, Tenth Revision* (*ICD-10*) diagnostic codes; *Current Procedural Terminology* codes; filled prescriptions; hospital-administered medications; and demographic information. As this study involved the analysis of preexisting, deidentified data, it was deemed exempt from requiring institutional review board approval by the University of Washington institutional review board. The need to obtain patient consent was waived by the University of Washington institutional review board because the Merative Marketscan Databases are deidentified. This cohort study adhered to the Strengthening the Reporting of Observational Studies in Epidemiology (STROBE) reporting guideline for reporting of observational studies.^10^

### Study Population

The population of interest consisted of individuals aged 18 to 64 years with RA who were identified using at least 2 RA *ICD-9* or *ICD-10* diagnostic codes 31 to 364 days apart (positive predictive value, 86.2%-88.9% for RA),^11^ on or before the date the patient initiated a TNFi, non-TNFi, or JAKi (index date). The study period was November 1, 2012 (when tofacitinib was first approved by the US Food and Drug Administration [FDA] for the treatment of RA^12^), through December 31, 2021.

Patients aged 65 years or older were excluded from our study, as this age group is predominantly covered by Medicare, and we did not have access to those claims. Patients with a diagnosis of HIV, congenital immunodeficiency, organ transplant, axial spondyloarthritis, psoriasis, or inflammatory bowel disease within 12 months prior to the index date were excluded to preserve a homogenous cohort due to their receiving similar treatments as patients with RA. Those with any prior cancer diagnoses, except NMSC, were excluded due to the difficulty and uncertainty of attributing cancer recurrence to a natural progression of disease vs a drug-induced adverse effect. Individuals with fewer than 90 days of follow-up were excluded because we hypothesized that cancers associated with the medications of interest were likely to take greater than 90 days to arise.

### Exposures of Interest

**Figure 1.**
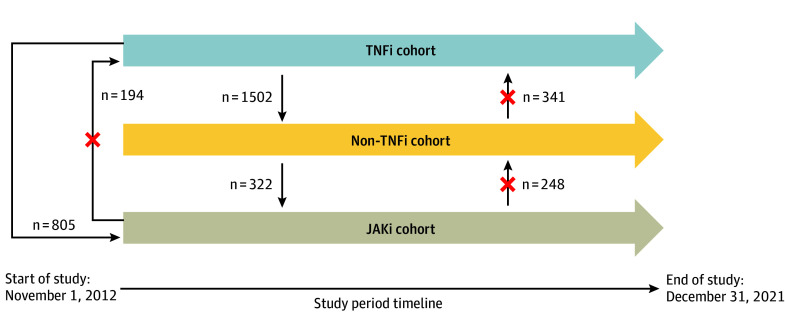
Study Design and Directions of Drug Class Switching JAKi indicates Janus kinase inhibitor; TNFi, tumor necrosis factor inhibitor.

We compared multiple treatment exposures: new initiations of TNFis (adalimumab, certolizumab, etanercept, golimumab, and infliximab), abatacept, IL-6is (tocilizumab and sarilumab), rituximab, or JAKis (tofacitinib, baricitinib, and upadacitinib). An individual could contribute person-time to more than 1 treatment exposure if treatment escalation mimicked typical clinical practice^13^ (Figure 1).

### Outcomes

The primary outcome of interest was any incident cancer, including breast, myeloid or lymphoid, lung, prostate, genitourinary, and colorectal cancer, identified using at least 1 *ICD-9* or *ICD-10* code by validated administrative algorithms after at least 90 days and within 2 years of treatment exposure, excluding NMSC (sensitivity, 40%-90%; specificity, ≥98%).^14^ Prior to analyzing our data, we decided to associate outcomes with the most recent biologic or targeted synthetic DMARD exposure, regardless of time after initiation.^15^

Follow-up ended when (1) the person switched drug categories, (2) filled more than 1 drug category on the same day, (3) did not have a prescription fill of a biologic or targeted synthetic DMARD for more than 90 days or more than 180 days for rituximab due to its longer washout period, (4) enrollment ended, or (5) receipt of a cancer diagnosis.

### Covariates

Multiple confounders that we hypothesized would be associated with treatment and cancer were identified a priori, including demographic characteristics (age, sex, geographic regions designated by the database [Northeast, North Central, South, West, and unknown], and year of initiating biologic), proxies of RA severity (glucocorticoid and conventional synthetic DMARD prescription fills and number of biologic drug switches), clinical characteristics (number of days from RA diagnosis to biologic or targeted synthetic DMARD initiation, tobacco use, frailty status,^16,17^ and Charlson comorbidity score^18^), and health care utilization within 12 months prior to starting treatment (hospital admissions, emergency department visits, outpatient visits, and opioid or nonsteroidal anti-inflammatory drug [NSAID] prescription fills).

### Statistical Analysis

Statistical analysis took place from June 2022 to September 2024. We calculated descriptive statistics stratified by drug treatment category and by whether people received a diagnosis of cancer or were censored. We also calculated the number of incident cancer diagnoses, incidence rates of cancer, and median number of days to incident cancer for each treatment exposure. To assess the risk of incident cancer by treatment exposure, we used Cox proportional hazards regression models adjusted for potential confounders to estimate hazard ratios (HRs) and 95% CIs.^19^ Tumor necrosis factor inhibitors served as the referent group because they are often the initial biologic used for RA refractory to conventional synthetic DMARD treatment.^1,2,3,4,5^ Uncontrolled inflammation has been shown to be a risk factor, especially for lymphoma, in RA. Inherently, a patient taking only methotrexate for RA is more likely to be an individual with less-active disease compared with a patient who requires biologic or targeted synthetic DMARDs to control their disease. In this study, we wanted to compare groups with relatively similar disease activity as much as possible to mitigate confounding by indication; therefore, we designated the TNFi group, rather than exposure to methotrexate or conventional synthetic DMARDs alone, as the referent.

We assessed the proportional hazards assumption by examining the *P* values of time-dependent explanatory variables and by examining plots of Schoenfeld residuals and determined that the proportional hazards assumption was satisfied. Corresponding Cox proportional hazard survival curves for time to incident cancer were generated for each treatment exposure, adjusted for confounders. A 2-sided *P* < .05 was considered statistically significant.

To address the potential for confounding by indication, we also conducted sensitivity analyses in which we used propensity matching to create 4 matched cohorts comparing cancer rates in people who were treated with rituximab, IL-6is, abatacept, and JAKis vs matched reference populations of those treated with TNFis. For each cohort, we matched 1:1 on each of the demographic characteristics, proxies of RA severity, clinical characteristics, and health care utilization metrics listed. We then conducted the same statistical analyses as in the primary analysis to obtain crude and adjusted incidence rates of cancer per 10 000 person-years as well as adjusted HRs and 95% CIs. We assessed whether the cohorts were well matched using standardized mean differences. We also evaluated whether increases in cancer awareness or detection changed over the calendar time of our study by evaluating the number of cancer events per 10 000 person-years from 2012 to 2016 compared with 2017 to 2021. Finally, because the molecular profiles of the 3 medications included in the JAKi category differ, we evaluated how many people were treated with tofacitinib, baricitinib, and upadacitinib and how many people treated with each medication developed cancer.

## Results

**Figure 2.**
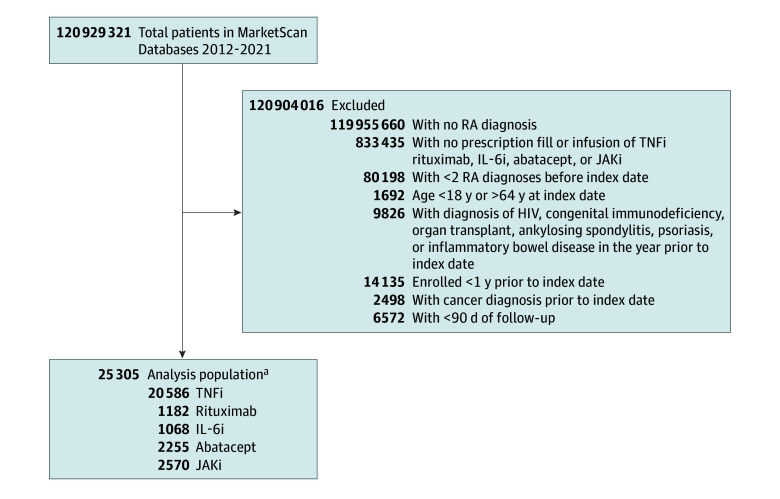
Study Flow Diagram IL-6i indicates interleukin 6 inhibitor; JAKi, Janus kinase inhibitor; RA, rheumatoid arthritis; and TNFi, tumor necrosis factor inhibitor. ^a^Patients could have taken more than 1 drug class, so the total sums to more than 25 305.

Of the 25 305 individuals who initiated treatment initiations and who met our inclusion criteria, 19 869 (79%) were female and 5436 (21%) were male (median age, 50 years [IQR, 42-56 years]), and 12 516 (49%) were from the South US (Table 1). Of a total 27 661 drug exposures, drug initiations consisted of 20 586 TNFi exposures (74%), 2570 JAKi exposures (9%), 2255 abatacept exposures (8%), 1182 rituximab exposures (4%), and 1068 IL-6i exposures (4%) (Figure 2). Treatment exposures were similar across geographic regions. Although the median year of biologic or targeted synthetic DMARD initiation was 2016 for most medication exposures, the JAKi cohorts began later in 2019, consistent with FDA approval of tofacitinib and baricitinib for use in RA.

**Table 1.** Baseline RA Cohort Characteristics and Covariates by Drug Class

Variable	Patients, No. (%)				
	TNFi (n = 20 586 [74%])	Rituximab (n = 1182 [4%])	IL-6i (n = 1068 [4%])	Abatacept (n = 2255 [8%])	JAKi (n = 2570 [9%])
Demographic characteristics					
Age, median (IQR), y	50 (41-55)	50 (41-55)	50 (42-56)	50 (43-56)	50 (43-56)
Sex					
Female	16 057 (78)	942 (80)	839 (79)	1904 (84)	2077 (81)
Male	4529 (22)	240 (20)	229 (21)	351 (16)	493 (19)
Geographic region					
Northeast	2723 (13)	201 (17)	147 (14)	314 (14)	395 (15)
North Central	4212 (20)	244 (21)	215 (20)	437 (19)	474 (18)
South	10 244 (50)	522 (44)	505 (47)	1130 (50)	1314 (51)
West	3118 (15)	201 (17)	187 (18)	346 (15)	375 (15)
Unknown	289 (1)	14 (1)	14 (1)	28 (1)	12 (1)
Year of treatment exposure, median (IQR)	2016 (2014-2018)	2016 (2014-2018)	2016 (2014-2018)	2016 (2014-2018)	2019 (2016-2020)
Proxies of RA severity					
Fills of glucocorticoids in 3 mo prior	9573 (47)	539 (46)	481 (45)	978 (43)	1091 (42)
Fills of conventional synthetic DMARD in 3 mo prior	14 825 (72)	536 (45)	540 (51)	1383 (61)	1703 (66)
Clinical characteristics					
Time from RA diagnosis to treatment exposure, median (IQR), d	470 (240-970)	680 (350-1310)	680 (380-1200)	640 (370-1210)	720 (400-1330)
Tobacco use	1555 (8)	97 (8)	76 (7)	169 (7)	176 (7)
Frailty score, median (IQR)^a^	0.13 (0.12-0.16)	0.14 (0.12-0.18)	0.14 (0.12-0.16)	0.14 (0.12-0.16)	0.13 (0.12-0.16)
Charlson comorbidity score					
1-2	18 157 (88)	897 (76)	887 (83)	1880 (83)	2258 (88)
3-4	1949 (9)	213 (18)	133 (12)	286 (13)	243 (9)
≥5	480 (2)	72 (6)	48 (4)	89 (4)	69 (3)
Health care utilization in 12 mo prior					
≥1 Hospital admission	1678 (8)	249 (21)	120 (11)	223 (10)	201 (8)
≥1 Emergency department visit	5408 (26)	458 (38)	340 (32)	671 (30)	609 (24)
≥1 Outpatient visit	20 574 (100)	1182 (100)	1068 (100)	2255 (100)	2567 (100)
≥1 Opioid prescription fill	8042 (39)	443 (37)	424 (40)	919 (41)	877 (34)
≥1 NSAID prescription fill	11 496 (56)	419 (35)	508 (48)	1156 (51)	1440 (56)
Reason for end of follow-up					
Enrollment ended	8030 (39)	247 (21)	341 (32)	842 (37)	1057 (41)
Did not refill in 90 of 180 d	10 278 (50)	913 (77)	474 (44)	869 (39)	1122 (44)
Switched to allowed drug category	2114 (10)	4 (0.3)	109 (10)	222 (10)	0
Switched to not allowed drug category	0	4 (0.3)	136 (13)	292 (13)	369 (14)
Filled >1 drug category on same day	2 (0.01)	0	0	0	0
Cancer outcome	162 (1)	14 (1)	8 (1)	30 (1)	22 (1)
Follow-up, median (IQR), d	300 (180-550)	190 (180-240)	230 (150-410)	260 (170-520)	270 (170-450)

Abbreviations: DMARD, disease-modifying antirheumatic drug; IL-6i, interleukin-6 inhibitor; JAKi, Janus kinase inhibitor; NSAID, nonsteroidal anti-inflammatory drug; RA, rheumatoid arthritis; TNFi, tumor necrosis factor inhibitor.

^a^ Frailty score: range, 0 (not at all frail) to 1 (severely frail).

Regarding proxies of RA severity, glucocorticoid prescription fills 3 months prior to study index were similar for all treatment exposures (JAKis, 42%; abatacept, 43%; IL-6is, 45%; rituximab, 46%; TNFis, 47%) (Table 1). The TNFi cohort had the shortest median duration from RA diagnosis to treatment exposure at 470 days (IQR, 240-970 days); the median of the other exposure groups ranged from 640 days (IQR, 370-1210 days) for abatacept to 720 days (IQR, 400-1330 days) for JAKis. Tobacco use, frailty score, and comorbidities were similar across treatment exposure groups, except for rituximab users, who had the largest proportion of moderate to severe comorbidities (24% with Charlson comorbidity scores of ≥3).

As for health care utilization in the year prior to treatment exposure, rituximab exposures had the highest percentage of hospital admissions (21%) and emergency department visits (38% compared with the other biologic or targeted synthetic DMARDs) (Table 1). However, rituximab also had the lowest percentage of NSAID prescription fills (35%). Otherwise, those initiating biologic or targeted synthetic DMARD therapies demonstrated similar percentages for these covariates, as well as opioid prescription fills (JAKis, 34%; rituximab, 37%; TNFIs, 39%; IL-6is, 40%; abatacept, 41%). The TNFi group had the highest median number of days of follow-up (300 days [IQR, 180-550 days]), while rituximab had the lowest (190 days [IQR, 180-240 days]). Most patients continued to receive the same class of biologic or targeted synthetic DMARDs throughout the duration of our study (TNFis, 90%; rituximab, 87%; JAKis, 65%; abatacept, 59%; IL-6is, 50%) (eTable 1 in Supplement 1).

Tumor necrosis factor inhibitor, IL-6i, and rituximab exposures were associated with the fewest median number of days to any incident cancer diagnosis (TNFis, 245 days [IQR, 152-437 days]; IL-6is, 230 days [IQR, 199-299 days]; and rituximab, 202 days [IQR, 157-303 days]), while abatacept and JAKis had the most number of median days (abatacept, 289 days [IQR, 150-373 days] and JAKis, 276 days [IQR, 169-351 days]) (Table 2).^16,17,18^ Rituximab exposures had the highest incidence rate (171 [95% CI, 94-285] cancer diagnoses per 10 000 person-years) compared with all other groups, followed by abatacept (142 [95% CI, 96-201] diagnoses per 10 000 person-years), JAKis (94 [95% CI, 59-143] diagnoses per 10 000 person-years), and IL-6is (88 [95% CI, 38-173] diagnoses per 10 000 person-years) compared with TNFis (78 [95% CI, 66-91] diagnoses per 10 000 person-years). A breakdown of cancer diagnoses by type is shown in eTable 2 in Supplement 1. When we compared incidence rates of cancer in the earlier (2012-2016) vs the later (2017-2021) years of our study, we found 87 (95% CI, 73-103) cancers per 10 000 person-years in the earlier years vs 88 (95% CI, 72-106) cancers per 10 000 person-years in the later years. When we examined the number of people who were treated with each of the 3 medications included in the JAKi category, we found 2117 of 2570 people (82%) were treated with tofacitinib, 437 of 2570 people (17%) were treated with upadacitinib, and 24 of 2570 people (1%) were treated with baricitinib. Of the 22 people in the JAKi category who received a diagnosis of cancer, 19 were treated with tofacitinib, 3 were treated with upadacitinib, and none were treated with baricitinib.

**Table 2.** Median Time to Cancer Diagnosis, Number of Incident Cancer Diagnoses per 10 000 Person-Years at Risk, and Adjusted HR After at Least 90 Days and Within 2 Years of Treatment Exposure by Drug Category

Biologic or targeted synthetic DMARD	No. (%) (N = 27 661)	Time to cancer diagnosis, median (IQR), d	Crude No. (%) of cancer diagnoses	No. of cancer diagnoses/10 000 person-years (95% CI)	Adjusted HR (95% CI)^a^
TNFi	20 586 (74)	245 (152-437)	162 (0.8)	78 (66-91)	1.00 [Reference]
Rituximab	1182 (4)	202 (157-303)	14 (1.2)	171 (94-285)	1.91 (1.17-3.14)
IL-6i	1068 (4)	230 (199-299)	8 (0.8)	88 (38-173)	1.04 (0.57-1.92)
Abatacept	2255 (8)	289 (150-373)	30 (1.3)	142 (96-201)	1.47 (1.03-2.11)
JAKi	2570 (9)	276 (169-351)	22 (0.9)	94 (59-143)	1.36 (0.94-1.96)

Abbreviations: DMARD, disease-modifying antirheumatic drug; HR, hazard ratio; IL-6i, interleukin 6 inhibitor; JAKi, Janus kinase inhibitor; RA, rheumatoid arthritis; TNFi, tumor necrosis factor inhibitor.

^a^ Models adjusted for individual covariates: demographics (age, sex, geographic region, and year of initiating biologic), proxies of RA severity (glucocorticoid and conventional synthetic disease-modifying antirheumatic drug prescription fills and number of drug switches), clinical characteristics (number of days from RA diagnosis to biologic initiation, tobacco use, frailty status,^16,17^ and Charlson comorbidity score^18^), and health care utilization within 12 months prior to starting treatment (hospital admissions, emergency department visits, outpatient visits, opioid or nonsteroidal anti-inflammatory drug prescription fills, and days of follow-up).

**Figure 3.**
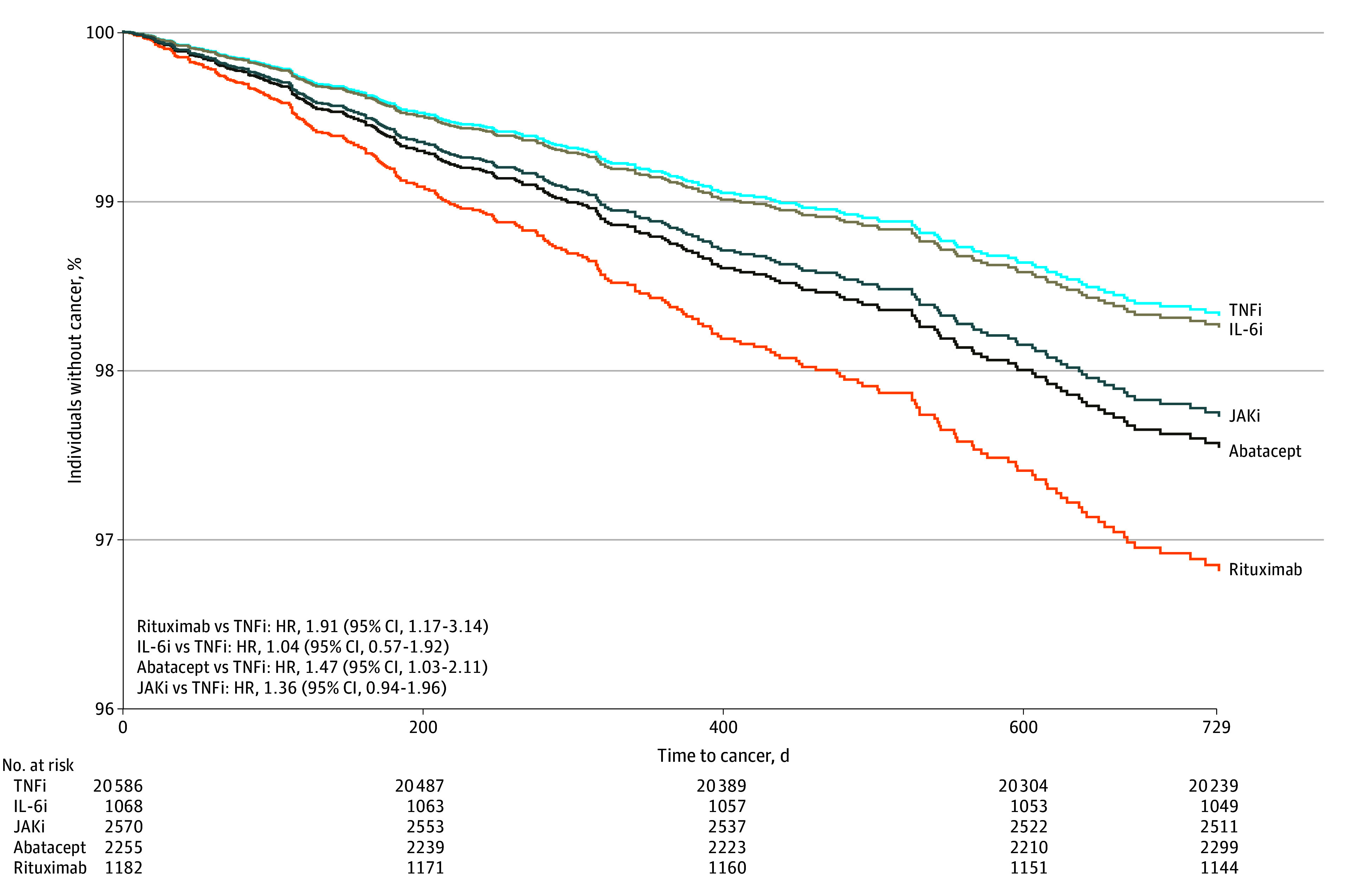
Survival Curves Generated From Multivariable Cox Proportional Hazards Regression Models for Time to Incident Cancer by Drug Class Exposure and Adjusted for Covariates Models adjusted for individual covariates: demographic characteristics (age, sex, geographic region, and year of initiating biologic), proxies of rheumatoid arthritis severity (glucocorticoid and conventional synthetic disease-modifying antirheumatic drug prescription fills and number of drug switches), clinical characteristics (number of days from rheumatoid arthritis diagnosis to biologic initiation, tobacco use, frailty status,^16,17^ and Charlson comorbidity score^18^), and health care utilization within 12 months prior to starting treatment (hospital admissions, emergency department visits, outpatient visits, opioid or nonsteroidal anti-inflammatory drug prescription fills, and number of days of follow-up). HR indicates hazard ratio, IL-6i, interleukin 6 inhibitor; JAKi, Janus kinase inhibitor; and TNFi, tumor necrosis factor inhibitor.

In the multivariable Cox proportional hazards regression analysis accounting for potential confounders, rituximab demonstrated a higher risk (HR, 1.91; 95% CI, 1.17-3.14) of any incident cancer compared with TNFis, followed by abatacept (HR, 1.47; 95% CI, 1.03-2.11) and JAKis (HR, 1.36; 95% CI, 0.94-1.96) (Table 2; Figure 3).^16,17,18^ Interleukin 6 inhibitors exposures had a similar risk of incident cancer as TNFis (HR, 1.04; 95% CI, 0.57-1.92).

In the propensity analysis examining associations between TNFis and each of the other drug treatments, we identified 4 well-matched cohorts (eTables 3, 5, 7, and 9 in Supplement 1), with no variables showing standardized mean differences greater than 0.10. Similar to the primary analysis, we found statistically significantly increased risk in those treated with rituximab (HR, 4.37; 95% CI, 1.48-12.93) (eTable 4 and eFigure 1 in Supplement 1) and abatacept (HR, 3.12; 95% CI, 1.52-6.44) (eTable 8 and eFigure 3 in Supplement 1). Also like the primary analysis, we found that those treated with JAKis did not have a statistically significant increase in risk of cancer (HR, 1.79; 95% CI, 0.91-3.56) (eTable 10 and eFigure 4 in Supplement 1). Although the primary analysis showed no association between treatment with IL-6is and cancer, we did observe a statistically significantly increased risk in the propensity-matched analysis (HR, 5.65; 95% CI, 1.11-28.79) (eTable 6 and eFigure 2 in Supplement 1). The small numbers of people with cancer in these propensity-matched analyses indicate that these results should be interpreted with caution.

When we examined descriptive statistics of the cohort stratified by whether they were censored vs received a diagnosis of cancer, we found that those who were censored tended to be younger and were more likely to have been female, had less severe RA, and had fewer comorbidities (eTable 11 in Supplement 1).

## Discussion

We observed that, among patients with RA, rituximab, abatacept, and JAKi initiations were associated with increased risks of incident cancer after 90 days and within 2 years of initiation, compared with TNFi initiation, although only rituximab and abatacept initiations had statistically significant associations. However, due to the relatively small overall number of incident cancer diagnoses, limited follow-up time for cancer occurrence, and potential residual confounding by indication, it is also possible that people who initiated non-TNFi (particularly rituximab and abatacept) and JAKi treatment are at higher risk of cancer, rather than the drugs themselves posing a higher risk.

We observed a significantly higher risk of incident cancer with rituximab compared with TNFi use (HR, 1.91). These findings conflict with rituximab’s mechanism of action as an anti-CD20 monoclonal antibody and B-cell–depleting agent that is also used for the treatment of B-cell lymphomas.^2,3,4,5,20,21^ Thus, we had hypothesized that there would be a lower incidence of cancer with rituximab compared with the other biologic or targeted synthetic DMARD exposures. Rituximab has also been studied in non-RA populations, including patients with multiple sclerosis, and was shown to not be associated with increased risk of cancer.^22^ Channeling bias is certainly possible, in that these individuals may have started treatment with rituximab out of concern for possible risk of a malignant neoplasm in addition to their RA diagnosis.

Furthermore, rituximab is used for managing refractory RA^1,2,3,4,5^ in individuals who tend to have more comorbidities and health care utilization (rituximab was associated with the highest percentage of moderate to severe Charlson comorbidity scores^18^ [24% for scores of ≥3], hospital admissions [21%], and emergency department visits [38%]). Such individuals may also be at higher risk for the outcome of interest (confounding by indication); thus, rituximab is likely not the factor associated with higher cancer incidence observed in this study.

Our findings of increased incidence rate and risk of cancer with JAKi exposure compared with TNFi exposure are consistent with the results of ORAL Surveillance,^6^ although the results were not statistically significant. Another cohort study using linked Swedish registries^8^ compared the risk of cancer development in patients with RA and psoriatic arthritis who had initiated JAKi, TNFi, or non-TNFi biologics.^8^ Similar to our study, they allowed for multiple treatment exposures per individual and required a lag time of 90 days after biologic or targeted synthetic DMARD initiation in which outcomes could not occur. Although they also showed no increased risk of most cancers for all drug classes, there was a statistically significantly increased risk of NMSC in patients receiving JAKis. More studies with longer follow-up are needed to better elucidate this finding.

Abatacept was associated with a higher risk of cancer compared with TNFis in our study. Prior studies have shown similar findings. A cohort study of patients with RA in the Consortium of Rheumatology Researchers of North America (CORRONA) registry (2001-2010) compared the risk of cancer development among new exposures of methotrexate (reference), nonbiologic DMARDs, TNFis, rituximab, and abatacept and found a numerically increased risk of cancer with abatacept exposures, although this finding was not statistically significant.^23^ An observational study of patients with RA identified using multiple databases from 2006 to 2014 found a higher risk of malignant neoplasms with abatacept initiations vs other DMARD therapies.^24^ Another cohort study using the Truven MarketScan Commercial and Supplement Medicare database also found an increased risk of cancer with new abatacept exposures.^25^

In contrast, using the World Health Organization’s VigiBase database of pharmacovigilance, new abatacept exposures were not associated with a higher risk of malignant neoplasms compared with other biologic DMARDs, although it did demonstrate a statistically significantly increased risk of melanoma diagnoses.^23^ Given various cohort and observational studies’ findings regarding abatacept’s cancer risk in treatment of rheumatologic disease, it is biologically plausible that abatacept is associated with an increased risk of cancer compared with other biologics since abatacept is a cytotoxic T-lymphocyte associated protein 4 (CTLA-4) agonist that directly opposes the mechanism of action of immunomodulators such as ipilimumab, a CTLA-4 antagonist.^26^ However, similar to rituximab, channeling bias is also possible, and prospective trials would be necessary to determine whether it increases cancer risk.

### Limitations

Our study has several limitations. We identified a relatively small number of cancer outcomes, which limited the ability to adjust for confounders. However, we were still able to detect statistically significant differences. We also followed the cohort for only a maximum of 2 years after treatment exposure for cancer development. Cancer risk was attributed to the most recent treatment exposure, irrespective of the duration between exposure and cancer diagnosis.^15^ Cancer outcomes occurring close to treatment initiation may be associated more with other pathophysiological processes, as cancer may require a longer latent period.

The claims data that we used also included only patients aged 18 to 64 years, so we are unable to generalize our findings to older individuals with RA. Because cancer incidence increases in older populations, this limitation may have partially explained our low number of outcomes overall. The use of US-specific claims data also limits our study’s generalizability to populations in other countries, given the socioeconomic, political, religious, and racial and ethnic differences between various nations.

Furthermore, confounding by indication must be considered. Inherently, individuals using non-TNFi biologic or targeted synthetic DMARDs likely had greater RA disease burden than those using TNFis, especially because most individuals with refractory RA initiate treatment with TNFis before proceeding to non-TNFI drugs or JAKis in clinical practice.^1,2,3,4,5^ These individuals are likely to have more comorbidities and increased risk of adverse outcomes. In addition, some individuals with RA may have started treatment with rituximab for a cancer indication but were incorrectly identified as not having cancer. This limitation reflects both confounding by indication as well as misclassification of the outcome and may lead to bias in our results. Although we attempted to account for these potential confounders, there may be unmeasured or residual confounding.

In addition, claims data do not have direct measures of RA disease activity or severity. To attempt to account for this, we used surrogate measures of RA disease burden—the use of glucocorticoids or conventional synthetic DMARDs—but we did not have access to other potentially explanatory data, such as genetic risk for cancer. Treatment misclassification is possible, as we did not have information on how well the patients adhered to their medications.

## Conclusions

In this cohort study of individuals with RA and new biologic or targeted synthetic DMARD exposures, we found that new rituximab and abatacept initiators demonstrated higher incidence rates and statistically significantly increased risks of incident cancers compared with TNFi initiators in the first 2 years after biologic or targeted synthetic DMARD initiation. However, given the limitations of using private insurance claims data and confounding by indication, it is likely that these patients may have a higher disease burden, resulting in channeling bias. To understand these associations, larger studies with longer follow-up and more granular collection of data, including medication indications and RA disease activity measures, would be needed for better comparison of incident cancer risk among these drugs. Additional studies that can supplement observational pharmacoepidemiology studies include postmarketing surveillance studies, additional phase 4 clinical trials that provide head-to-head comparisons between JAKis and different biologics with intention-to-treat analyses, and meta-analyses comparing studies from US and international databases.

## Notes

Supplement 1. **eTable 1.** Crude Number and Percentages of Treatment Exposure Switches by Drug Class **eTable 2.** Number of Incident Cancer Diagnoses per 10,000 Person-Years at Risk (95% CI) by Cancer Type After at Least 90 Days and Within 2 Years of Treatment Exposure by Drug Category **eTable 3.** Baseline Characteristics of the Matched Cohort Comparing TNFi to Rituximab **eTable 4.** Median Days to Cancer Diagnosis, Number of Incident Cancer Diagnoses (95% CI) per 10,000 Person-Years at Risk, and Adjusted HR (95% CI) Comparing TNFi to Rituximab **eFigure 1.** Adjusted Cox Proportional Hazards Curves for Time to Incident Cancer in Those Treated With TNFi Compared to Rituximab **eTable 5.** Baseline Characteristics of the Matched Cohort Comparing TNFi to Interleukin-6 Inhibitors (IL-6i) **eTable 6.** Median Days to Cancer Diagnosis, Number of Incident Cancer Diagnoses (95% CI) per 10,000 Person-Years at Risk, and Adjusted HR (95% CI) Comparing TNFi to Interleukin-6 Inhibitors (IL-6i) **eFigure 2.** Adjusted Cox Proportional Hazards Curves for Time to Incident Cancer in Those Treated With TNFi Compared to Interleukin-6 Inhibitors (IL-6i) **eTable 7.** Baseline Characteristics of the Matched Cohort Comparing TNFi to Abatacept **eTable 8.** Median Days to Cancer Diagnosis, Number of Incident Cancer Diagnoses (95% CI) per 10,000 Person-Years at Risk, and Adjusted HR (95% CI) Comparing TNFi to Abatacept **eFigure 3.** Adjusted Cox Proportional Hazards Curves for Time to Incident Cancer in Those Treated With TNFi Compared to Abatacept **eTable 9.** Baseline Characteristics of the Matched Cohort Comparing TNFi to JAKi **eTable 10.** Median Days to Cancer Diagnosis, Number of Incident Cancer Diagnoses (95% CI) per 10,000 Person-Years at Risk, and Adjusted HR (95% CI) Comparing TNFi to JAKi **eFigure 4.** Adjusted Cox Proportional Hazards Curves for Time to Incident Cancer in Those Treated With TNFi Compared to JAKi **eTable 11.** Baseline Characteristics Comparing Those Who Were Censored to Those Who Were Diagnosed With Cancer

Supplement 2.
Data Sharing Statement
