# Supplementary material for: Use of Biologic or Targeted Synthetic Disease-Modifying Antirheumatic Drugs and Cancer Risk
Source: JAMA Netw Open. 2024 Nov 20;7(11):e2446336. doi: 10.1001/jamanetworkopen.2024.46336 (PMC11579790; doi:10.1001/jamanetworkopen.2024.46336)
Supplement: Supplement 1. — eTable 1. Crude Number and Percentages of Treatment Exposure Switches by Drug Class eTable 2. Number of Incident Cancer Diagnoses per 10,000 Person-Years at Risk (95% CI) by Cancer Type After at Least 90 Days and Within 2 Years of Treatment Exposure by Drug Category eTable 3. Baseline Characteristics of the Matched Cohort Comparing TNFi to Rituximab eTable 4. Median Days to Cancer Diagnosis, Number of Incident Cancer Diagnoses (95% CI) per 10,000 Person-Years at Risk, and Adjusted HR (95% CI) Comparing TNFi to Rituximab eFigure 1. Adjusted Cox Proportional Hazards Curves for Time to Incident Cancer in Those Treated With TNFi Compared to Rituximab eTable 5. Baseline Characteristics of the Matched Cohort Comparing TNFi to Interleukin-6 Inhibitors (IL-6i) eTable 6. Median Days to Cancer Diagnosis, Number of Incident Cancer Diagnoses (95% CI) per 10,000 Person-Years at Risk, and Adjusted HR (95% CI) Comparing TNFi to Interleukin-6 Inhibitors (IL-6i) eFigure 2. Adjusted Cox Proportional Hazards Curves for Time to Incident Cancer in Those Treated With TNFi Compared to Interleukin-6 Inhibitors (IL-6i) eTable 7. Baseline Characteristics of the Matched Cohort Comparing TNFi to Abatacept eTable 8. Median Days to Cancer Diagnosis, Number of Incident Cancer Diagnoses (95% CI) per 10,000 Person-Years at Risk, and Adjusted HR (95% CI) Comparing TNFi to Abatacept eFigure 3. Adjusted Cox Proportional Hazards Curves for Time to Incident Cancer in Those Treated With TNFi Compared to Abatacept eTable 9. Baseline Characteristics of the Matched Cohort Comparing TNFi to JAKi eTable 10. Median Days to Cancer Diagnosis, Number of Incident Cancer Diagnoses (95% CI) per 10,000 Person-Years at Risk, and Adjusted HR (95% CI) Comparing TNFi to JAKi eFigure 4. Adjusted Cox Proportional Hazards Curves for Time to Incident Cancer in Those Treated With TNFi Compared to JAKi eTable 11. Baseline Characteristics Comparing Those Who Were Censored to Those Who Were Diagnosed With Cancer [file jamanetwopen-e2446336-s001.pdf]

Supplementary Online Content

Sendaydiego X, Gold LS, Dubreuil M, et al. Use of biologic or targeted synthetic disease-modifying antirheumatic drugs and cancer risk. *JAMA Netw Open.* 2024;7(11):e2446336. doi:10.1001/jamanetworkopen.2024.46336

eTable 1. Crude Number and Percentages of Treatment Exposure Switches by Drug Class
eTable 2. Number of Incident Cancer Diagnoses per 10,000 Person-Years at Risk (95% CI) by Cancer Type After at Least 90 Days and Within 2 Years of Treatment Exposure by Drug Category
eTable 3. Baseline Characteristics of the Matched Cohort Comparing TNFi to Rituximab
eTable 4. Median Days to Cancer Diagnosis, Number of Incident Cancer Diagnoses (95% CI) per 10,000 Person-Years at Risk, and Adjusted HR (95% CI) Comparing TNFi to Rituximab
eFigure 1. Adjusted Cox Proportional Hazards Curves for Time to Incident Cancer in Those Treated With TNFi Compared to Rituximab
eTable 5. Baseline Characteristics of the Matched Cohort Comparing TNFi to Interleukin-6 Inhibitors (IL-6i)
eTable 6. Median Days to Cancer Diagnosis, Number of Incident Cancer Diagnoses (95% CI) per 10,000 Person-Years at Risk, and Adjusted HR (95% CI) Comparing TNFi to Interleukin-6 Inhibitors (IL-6i)
eFigure 2. Adjusted Cox Proportional Hazards Curves for Time to Incident Cancer in Those Treated With TNFi Compared to Interleukin-6 Inhibitors (IL-6i)
eTable 7. Baseline Characteristics of the Matched Cohort Comparing TNFi to Abatacept
eTable 8. Median Days to Cancer Diagnosis, Number of Incident Cancer Diagnoses (95% CI) per 10,000 Person-Years at Risk, and Adjusted HR (95% CI) Comparing TNFi to Abatacept
eFigure 3. Adjusted Cox Proportional Hazards Curves for Time to Incident Cancer in Those Treated With TNFi Compared to Abatacept
eTable 9. Baseline Characteristics of the Matched Cohort Comparing TNFi to JAKi
eTable 10. Median Days to Cancer Diagnosis, Number of Incident Cancer Diagnoses (95% CI) per 10,000 Person-Years at Risk, and Adjusted HR (95% CI) Comparing TNFi to JAKi
eFigure 4. Adjusted Cox Proportional Hazards Curves for Time to Incident Cancer in Those Treated With TNFi Compared to JAKi
eTable 11. Baseline Characteristics Comparing Those Who Were Censored to Those Who Were Diagnosed With Cancer

This supplementary material has been provided by the authors to give readers additional information about their work.

© 2024 Sendaydiego X et al. *JAMA Network Open.*

eTable 1. Crude Number and Percentages of Treatment Exposure Switches by Drug Class

**Variable**	**TNFi n = 20,586 (74%)**	**Rituximab n = 1,182 (4%)**	**IL-6i n = 1,068 (4%)**	**Abatacept n = 2,255 (8%)**	**JAKi n = 2,570 (9%)**
# Treatment exposure switches (n (%))					
Single drug class	18,457 (90)	1023 (87)	535 (50)	1322 (59)	1677 (65)
Switched 1 time	1850 (9)	127 (11)	361 (34)	710 (31)	751 (29)
Switched 2 times	248 (1)	27 (2)	148 (14)	195 (9)	120 (5)
Switched 3 times	31 (0)	4 (0)	24 (2)	28 (1)	21 (1)
Switched 4 times	0 (0)	1 (0)	0 (0)	0 (0)	1 (0)

© 2024 Sendaydiego X et al. *JAMA Network Open.*

eTable 2. Number of Incident Cancer Diagnoses per 10,000 Person-Years at Risk (95% CI) by Cancer Type After at Least 90 Days and Within 2 Years of Treatment Exposure by Drug Category

	TNFi n = 20,586 (74%)	Rituximab n = 1,182 (4%)	IL-6i n = 1,068 (4%)	Abatacept n = 2,255 (8%)	JAKi n = 2,570 (9%)	All Drugs
**Crude n (%) of cancer diagnoses**	162 (0.8)	14 (1.2)	8 (0.8)	30 (1.3)	22 (0.9)	236 (0.9)
**# Cancer/ 10,000 person-years (95% CI)**	78 (66-91)	171 (94-285)	88 (38-173)	142 (96-201)	94 (59-143)	87 (77-99)
**Crude n (%) of breast cancer**	46 (0.2)	2 (0.2)	1 (0.1)	6 (0.3)	8 (0.3)	63 (0.2)
**# Breast cancer/10,000 person-years (95% CI)**	39 (28-52)	1 (0 ,4)	1 (0-4)	5 (2-10)	7 (3-13)	52 (40-66)
**Crude n (%) of myeloid/lymphoid cancer**	22 (0.1)	8 (0.7)	4 (0.4)	8 (0.4)	3 (0.1)	45 (0.2)
**# Myeloid/lymphoid cancer/10,000 person-years (95% CI)**	18 (11-27)	5 (2-11)	2 (1-6)	6 (3-13)	2 (0-5)	34 (25-45)
**Crude n (%) of lung cancer**	14 (0.1)	0	0	1 (0)	4 (0.2)	19 (0.1)
**# Lung cancer/10,000 person-years (95% CI)**	14 (8-24)	0	0	1 (0-6)	5 (1-12)	20 (12-31)
**Crude n (%) of prostate cancer**	11 (0.1)	1 (0.1)	1 (0.1)	4 (0.2)	2 (0.1)	19 (0.1)
**# Prostate cancer/10,000 person-years (95% CI)**	10 (5-18)	1 (0-5)	2 (0-9)	3 (1-9)	2 (0-7)	18 (11-28)
**Crude n (%) of genitourinary cancer**	11 (0.1)	2 (0.2)	1 (0.1)	0	1 (0)	15 (0.1)
**# Genitourinary cancer/10,000 person-years (95% CI)**	10 (5-18)	1 (0-2)	1 (0-5)	0	0 (0-2)	12 (7-19)
**Crude n (%) of colorectal cancer**	9 (0)	0	1 (0.1)	1 (0)	1 (0)	11 (0)
**# Colorectal cancer/10,000 person-years (95% CI)**	8 (4-15)	0	1 (0-3)	4 (0-25)	2 (0-10)	10 (5-18)
**Crude n (%) of other cancer**	69 (0.3)	1 (0.1)	1 (0.1)	14 (0.6)	4 (0.2)	89 (0.3)
**# Other cancer/10,000 person-years (95% CI)**	56 (43-71)	1 (0-4)	1 (0-3)	11 (6-18)	2 (1-5)	70 (56-86)

© 2024 Sendaydiego X et al. *JAMA Network Open.*

eTable 3. Baseline Characteristics of the Matched Cohort Comparing TNFi to Rituximab

Variable (No. (%)) unless otherwise specified)	Matched		
	**TNFi (n=1122)**	**Rituximab (n=1122)**	**Standardized Mean Difference**
**Demographics**			
Age in years (median (IQR))	49 (42-55)	49 (41-55)	0.01
Male	229 (20.4%)	220 (19.6%)	0.02
Female	893 (79.6%)	902 (80.4%)	
Geographic region			
Northeast	178 (15.9%)	183 (16.3%)	
North Central	221 (19.7%)	232 (20.7%)	0.00
South	552 (49.2%)	501 (44.7%)	
West	146 (13.0%)	192 (17.1%)	
Unknown	25 (2.2%)	14 (1.2%)	
Year of treatment exposure (median (IQR))	2016 (2014-2018)	2016 (2014-2018)	-0.05
**Proxies of RA severity**			
≥1 Fill of glucocorticoids in 3 months prior	500 (44.6%)	506 (45.1%)	0.01
≥1 Fill of csDMARD in 3 months prior	527 (47.0%)	531 (47.3%)	0.01
**Clinical characteristics**			
Days from RA diagnosis to treatment exposure (median (IQR))	570 (300-1230)	660 (350-1270)	-0.01
Tobacco use	79 (7.0%)	90 (8.0%)	0.04
Frailty score (0 [not at all frail] to 1 [severely frail]) (median (IQR))	0.14 (0.12-0.17)	0.14 (0.12-0.17)	0.03
Charlson co-morbidity score			
1-2	894 (79.7%)	873 (77.8%)	
3-4	161 (14.3%)	198 (17.6%)	0.02
5+	67 (6.0%)	51 (4.5%)	
**Healthcare utilization in 12 months prior**			
≥1 Hospital admission	225 (20.1%)	206 (18.4%)	-0.05
≥1 Emergency department visit	416 (37.1%)	416 (37.1%)	0.00
≥1 Outpatient visit	1,122 (100%)	1,122 (100%)	0.01
≥1 Opioid prescription fill	408 (36.4%)	418 (37.3%)	0.02
≥1 NSAID prescription fill	431 (38.4%)	409 (36.5%)	-0.04
**Reason for end of follow-up**			
Enrollment ended	424 (37.8%)	236 (21.0%)	
Did not refill in 90/180 days	521 (46.4%)	865 (77.1%)	
Switched to allowed drug category	172 (15.3%)	6 (0.5%)	
Swithed to not allowed drug category	0	2 (0.2%)	
Cancer Outcome	5 (0.5%)	13 (1.2%)	
**Days of follow-up (median (IQR))**	290 (170-510)	190 (180-260)	

© 2024 Sendaydiego X et al. *JAMA Network Open.*

eTable 4. Median Days to Cancer Diagnosis, Number of Incident Cancer Diagnoses (95% CI) per 10,000 Person-Years at Risk, and Adjusted HR (95% CI) Comparing TNFi to Rituximab

	**Median (IQR) days to cancer diagnosis**	**n (%) of cancer diagnoses**	**# of cancer diagnoses/ 10,000 person-years (95% CI)**	**Adjusted* HR (95% CI)**
TNFi	172 (139-204)	5 (0.5%)	46 (15-107)	Referent
Rituximab	221 (157-303)	13 (1.2%)	166 (89-282)	4.4 (1.5, 12.8)

* Adjusted for demographics (age, sex, region, year of initiating biologic), proxies of RA severity (glucocorticoid and csDMARDs prescription fills and number of drug switches), clinical characteristics (days from RA diagnosis to biologic initiation, tobacco use, frailty status, Charlson comorbidity score), and healthcare utilization in the 12 months prior to starting treatment (hospital admissions, emergency department visits, outpatient visits, opioid fills, and NSAID fills).

© 2024 Sendaydiego X et al. *JAMA Network Open.*

eFigure 1. Adjusted Cox Proportional Hazards Curves for Time to Incident Cancer in Those Treated With TNFi Compared to Rituximab

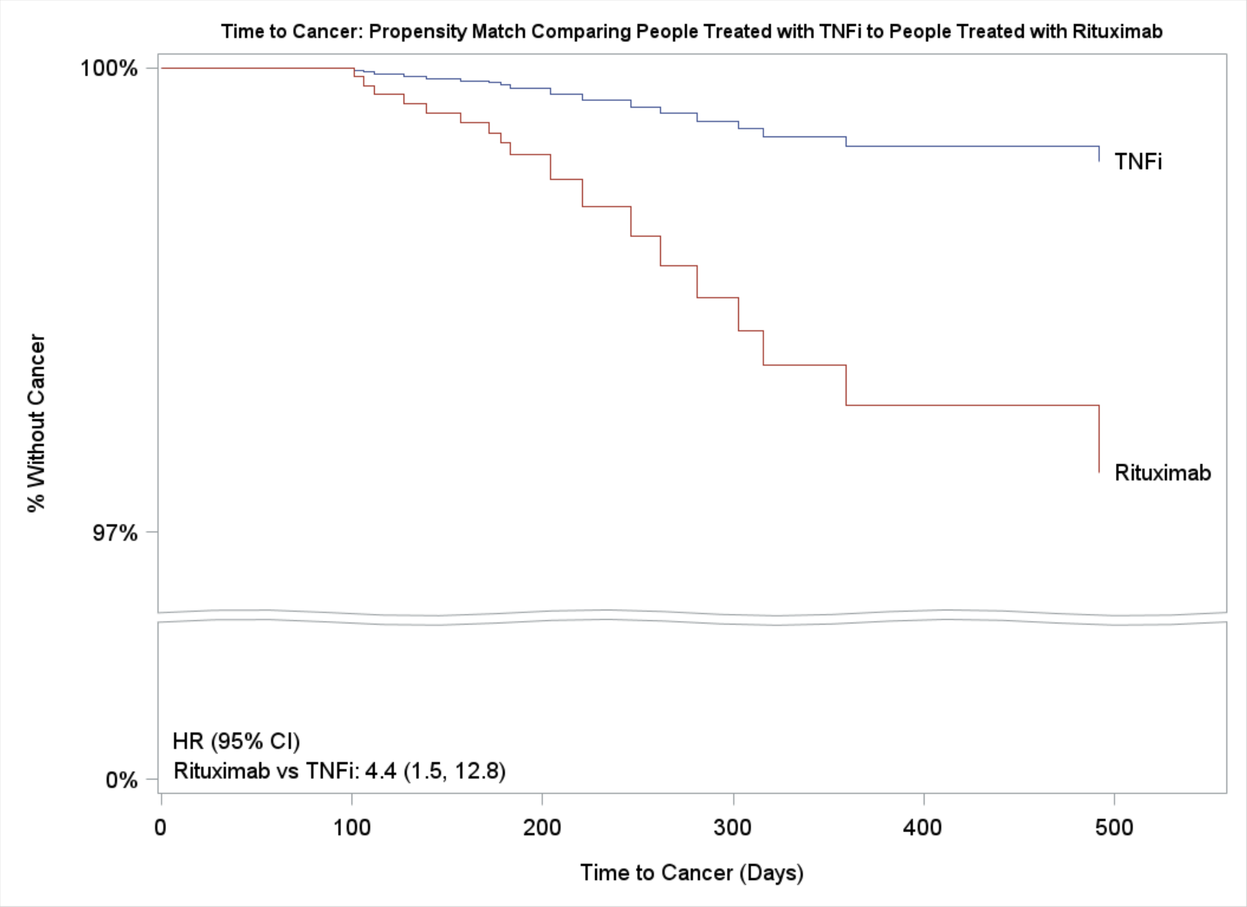

© 2024 Sendaydiego X et al. *JAMA Network Open.*

eTable 5. Baseline Characteristics of the Matched Cohort Comparing TNFi to Interleukin-6 Inhibitors (IL-6i)

Variable (No. (%)) unless otherwise specified)	Matched		
	**TNFi (n=977)**	**Interleukin-6 inhibitors (n=977)**	**Standardized Mean Difference**
**Demographics**			
Age in years (median (IQR))	50 (42-56)	50 (42-56)	0.00
Male	185 (18.9%)	205 (21.0%)	-0.05
Female	792 (81.1%)	772 (79.0%)	
Geographic region			
Northeast	115 (11.8%)	138 (14.1%)	
North Central	191 (19.5%)	199 (20.4%)	-0.03
South	519 (53.1%)	458 (46.9%)	
West	136 (13.9%)	168 (17.2%)	
Unknown	16 (1.6%)	14 (1.4%)	
Year of treatment exposure (median (IQR))	2016 (2014-2019)	2016 (2014-2018)	-0.02
**Proxies of RA severity**			
≥1 Fill of glucocorticoids in 3 months prior	422 (43.2%)	436 (44.6%)	0.03
≥1 Fill of csDMARD in 3 months prior	468 (47.9%)	510 (52.2%)	0.09
**Clinical characteristics**			
Days from RA diagnosis to treatment exposure (median (IQR))	560 (250-1270)	650 (370-1190)	-0.04
Tobacco use	58 (5.9%)	71 (7.3%)	0.05
Frailty score (0 [not at all frail] to 1 [severely frail]) (median (IQR))	0.14 (0.12-0.17)	0.14 (0.12-0.16)	-0.02
Charlson co-morbidity score			
1-2	832 (85.2%)	814 (83.3%)	-0.02
3-4	89 (9.1%)	122 (12.5%)	
5+	56 (5.7%)	41 (4.2%)	
**Healthcare utilization in 12 months prior**			
≥1 Hospital admission	105 (10.7%)	108 (11.1%)	0.01
≥1 Emergency department visit	309 (31.6%)	305 (31.2%)	-0.01
≥1 Outpatient visit	977 (100%)	977 (100%)	-0.03
≥1 Opioid prescription fill	375 (38.4%)	386 (39.5%)	0.02
≥1 NSAID prescription fill	454 (46.5%)	472 (48.3%)	0.04
**Reason for end of follow-up**			
Enrollment ended	234 (24.0%)	333 (34.1%)	
Did not refill in 90/180 days	320 (32.8%)	452 (46.3%)	
Switched to allowed drug category	421 (43.1%)	142 (14.5%)	
Swithed to not allowed drug category	0	42 (4.3%)	
Cancer Outcome	2 (0.2%)	8 (0.8%)	
Days of follow-up (median (IQR))	280 (170-480)	240 (150-420)	

© 2024 Sendaydiego X et al. *JAMA Network Open.*

eTable 6. Median Days to Cancer Diagnosis, Number of Incident Cancer Diagnoses (95% CI) per 10,000 Person-Years at Risk, and Adjusted HR (95% CI) Comparing TNFi to Interleukin-6 Inhibitors (IL-6i)

	**Median (IQR) days to cancer diagnosis**	**n (%) of cancer diagnoses**	**# of cancer diagnoses/ 10,000 person-years (95% CI)**	**Adjusted* HR (95% CI)**
TNFi	242 (208-276)	2 (0.2%)	22 (3-78)	Referent
Interleukin-6 inhibitors (IL-6i)	231 (199-299)	8 (0.8%)	96 (41-188)	5.7 (1.1-28.8)

* Adjusted for demographics (age, sex, region, year of initiating biologic), proxies of RA severity (glucocorticoid and csDMARDs prescription fills and number of drug switches), clinical characteristics (days from RA diagnosis to biologic initiation, tobacco use, frailty status, Charlson comorbidity score), and healthcare utilization in the 12 months prior to starting treatment (hospital admissions, emergency department visits, outpatient visits, opioid fills, and NSAID fills).

© 2024 Sendaydiego X et al. *JAMA Network Open.*

eFigure 2. Adjusted Cox Proportional Hazards Curves for Time to Incident Cancer in Those Treated With TNFi Compared to Interleukin-6 Inhibitors (IL-6i)

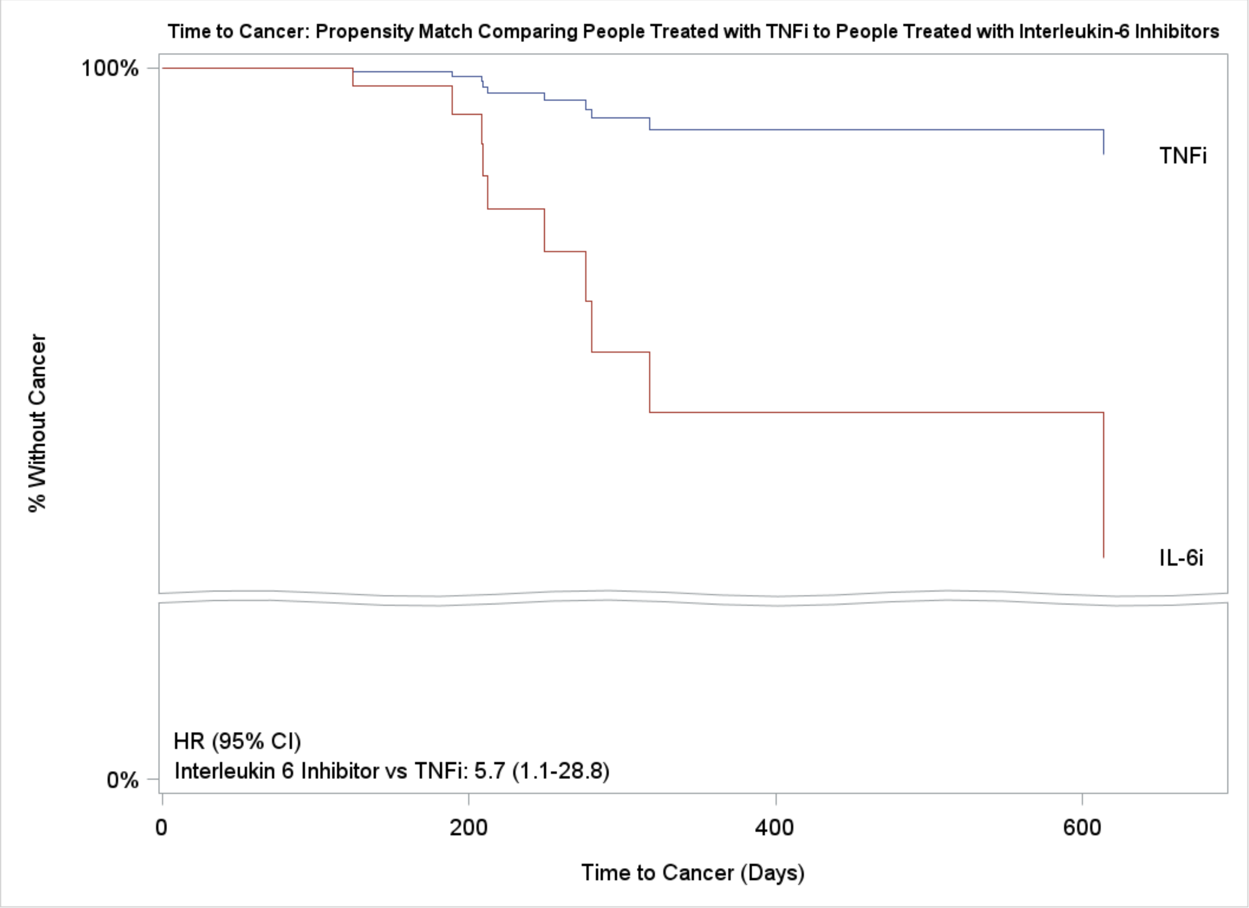

© 2024 Sendaydiego X et al. *JAMA Network Open.*

eTable 7. Baseline Characteristics of the Matched Cohort Comparing TNFi to Abatacept

**Variable (No. (%)) unless otherwise specified)**	**Matched**		
	**TNFi (n=2143)**	**Abatacept (n=2143)**	**Standardized Mean Difference**
**Demographics**			
Age in years (median (IQR))	50 (43-55)	50 (43-56)	0.01
Male	306 (14.3%)	340 (15.9%)	
Female	1,837 (85.7%)	1,803 (84.1%)	-0.04
Geographic region			
Northeast	268 (12.5%)	301 (14.0%)	
North Central	431 (20.1%)	410 (19.1%)	-0.02
South	1,097 (51.2%)	1,069 (49.9%)	
West	318 (14.8%)	335 (15.6%)	
Unknown	29 (1.4%)	28 (1.3%)	-0.05
Year of treatment exposure (median (IQR))	2016 (2014-2018)	2016 (2014-2018)	
**Proxies of RA severity**			
≥1 Fill of glucocorticoids in 3 months prior	900 (42.0%)	931 (43.4%)	0.03
≥1 Fill of csDMARD in 3 months prior	1,283 (59.9%)	1,322 (61.7%)	0.04
**Clinical characteristics**			
Days from RA diagnosis to treatment exposure (median (IQR))	540 (260-1270)	640 (360-1180)	-0.03
Tobacco use	145 (6.8%)	160 (7.5%)	0.03
Frailty score (0 [not at all frail] to 1 [severely frail]) (median (IQR))	0.14 (0.12-0.16)	0.14 (0.12-0.16)	-0.03
Charlson co-morbidity score			-0.02
1-2	1,797 (83.9%)	1,794 (83.7%)	
3-4	247 (11.5%)	273 (12.7%)	
5+	99 (4.6%)	76 (3.5%)	
**Healthcare utilization in 12 months prior**			
≥1 Hospital admission	220 (10.3%)	212 (9.9%)	-0.01
≥1 Emergency department visit	654 (30.5%)	634 (29.6%)	-0.02
≥1 Outpatient visit	2,142 (100%)	2,143 (100%)	-0.02
≥1 Opioid prescription fill	835 (39.0%)	868 (40.5%)	0.03
≥1 NSAID prescription fill	1,085 (50.6%)	1,097 (51.2%)	0.01
**Reason for end of follow-up**			
Enrollment ended	601 (28.0%)	831 (38.8%)	
Did not refill in 90/180 days	765 (35.7%)	851 (39.7%)	
Switched to allowed drug category	767 (35.8%)	305 (14.2%)	
Swithed to not allowed drug category	0	126 (5.9%)	
Cancer Outcome	10 (0.5%)	30 (1.4%)	
Days of follow-up (median (IQR))	290 (180-530)	260 (170-520)	

© 2024 Sendaydiego X et al. *JAMA Network Open.*

eTable 8. Median Days to Cancer Diagnosis, Number of Incident Cancer Diagnoses (95% CI) per 10,000 Person-Years at Risk, and Adjusted HR (95% CI) Comparing TNFi to Abatacept

	**Median (IQR) days to cancer diagnosis**	**n (%) of cancer diagnoses**	**# of cancer diagnoses/ 10,000 person-years (95% CI)**	**Adjusted* HR (95% CI)**
TNFi	196 (148-276)	10 (0.5%)	48 (23-87)	Referent
Abatacept	289 (150-373)	30 (1.4%)	148 (100-211)	3.1 (1.5-6.4)

* Adjusted for demographics (age, sex, region, year of initiating biologic), proxies of RA severity (glucocorticoid and csDMARDs prescription fills and number of drug switches), clinical characteristics (days from RA diagnosis to biologic initiation, tobacco use, frailty status, Charlson comorbidity score), and healthcare utilization in the 12 months prior to starting treatment (hospital admissions, emergency department visits, outpatient visits, opioid fills, and NSAID fills).

© 2024 Sendaydiego X et al. *JAMA Network Open.*

eFigure 3. Adjusted Cox Proportional Hazards Curves for Time to Incident Cancer in Those Treated With TNFi Compared to Abatacept

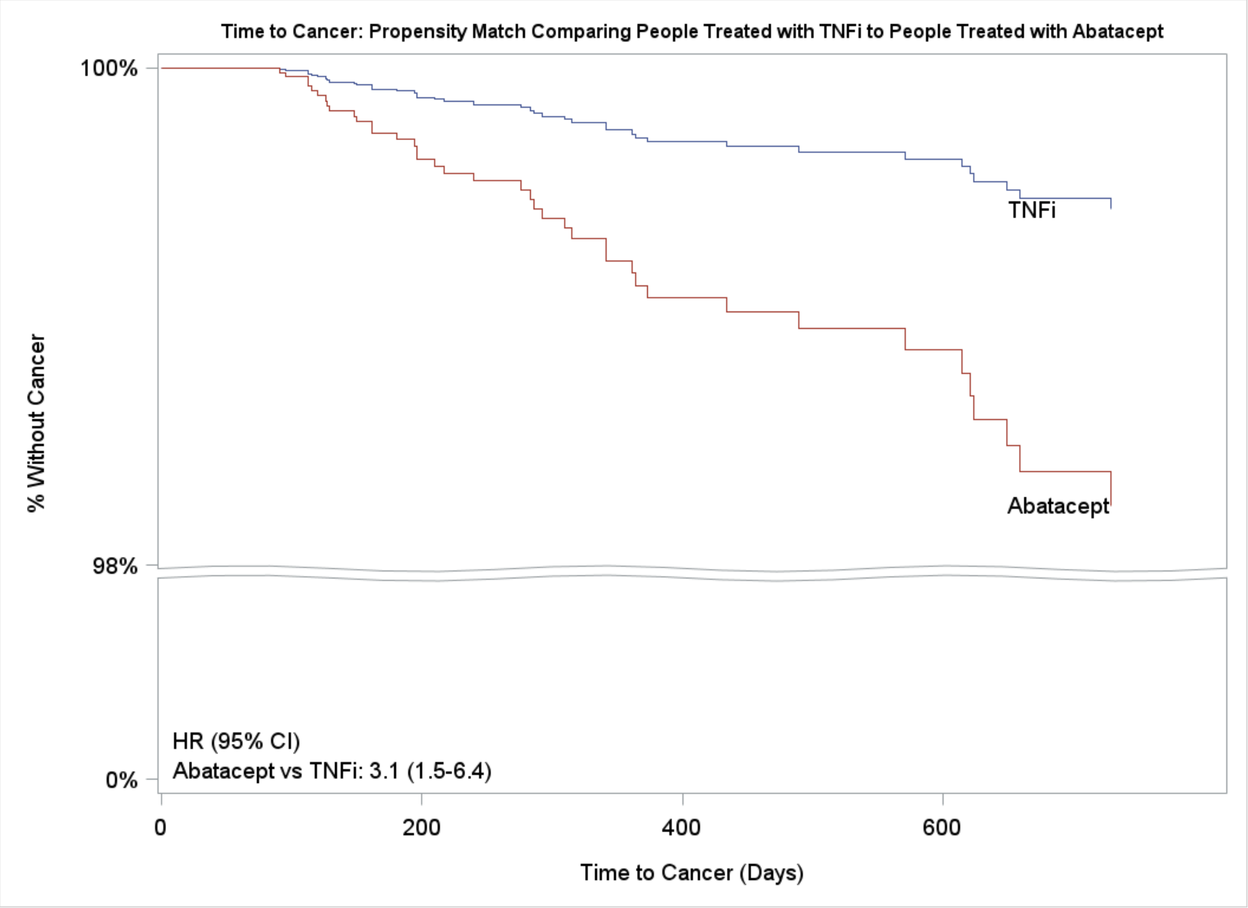

© 2024 Sendaydiego X et al. *JAMA Network Open.*

eTable 9. Baseline Characteristics of the Matched Cohort Comparing TNFi to JAKi

Variable (No. (%)) unless otherwise specified)	Matched		
	**TNFi (n=2421)**	**JAKi (n=2421)**	**Standardized Mean Difference**
**Demographics**			
Age in years (median (IQR))	50 (43-56)	50 (43-56)	-0.01
Male	463 (19.1%)	470 (19.4%)	-0.01
Female	1,958 (80.9%)	1,951 (80.6%)	
Geographic region			
Northeast	308 (12.7%)	376 (15.5%)	0.00
North Central	506 (20.9%)	438 (18.1%)	
South	1,304 (53.9%)	1,238 (51.1%)	
West	291 (12.0%)	358 (14.8%)	
Unknown	12 (0.5%)	11 (0.5%)	
Year of treatment exposure (median (IQR))	2018 (2016-2020)	2018 (2016-2020)	-0.01
**Proxies of RA severity**			
≥1 Fill of glucocorticoids in 3 months prior	1,040 (43.0%)	1,033 (42.7%)	-0.01
≥1 Fill of csDMARD in 3 months prior	1,644 (67.9%)	1,624 (67.1%)	-0.02
**Clinical characteristics**			
Days from RA diagnosis to treatment exposure (median (IQR))	580 (310-1270)	690 (390-1260)	0.01
Tobacco use	162 (6.7%)	169 (7.0%)	0.01
Frailty score (0 [not at all frail] to 1 [severely frail]) (median (IQR))	0.13 (0.12-0.16)	0.13 (0.12-0.16)	-0.03
Charlson co-morbidity score			
1-2	2,120 (87.6%)	2,121 (87.6%)	0.02
3-4	248 (10.2%)	232 (9.6%)	
5+	53 (2.2%)	68 (2.8%)	
Healthcare utilization in 12 months prior			
≥1 Hospital admission	190 (7.8%)	190 (7.8%)	0.00
≥1 Emergency department visit	603 (24.9%)	572 (23.6%)	-0.03
≥1 Outpatient visit	2,420 (100%)	2,420 (100%)	0.00
≥1 Opioid prescription fill	824 (34.0%)	824 (34.0%)	0.00
≥1 NSAID prescription fill	1,372 (56.7%)	1,352 (55.8%)	-0.02
**Reason for end of follow-up**			
Enrollment ended	908 (37.5%)	979 (40.4%)	
Did not refill in 90/180 days	851 (35.2%)	1084 (44.8%)	
Switched to allowed drug category	648 (26.8%)	176 (7.3%)	
Swithed to not allowed drug category	0	160 (6.6%)	
Cancer Outcome	14 (0.6%)	22 (0.9%)	
Days of follow-up (median (IQR))	290 (180-500)	270 (170-460)	

© 2024 Sendaydiego X et al. *JAMA Network Open.*

eTable 10. Median Days to Cancer Diagnosis, Number of Incident Cancer Diagnoses (95% CI) per 10,000 Person-Years at Risk, and Adjusted HR (95% CI) Comparing TNFi to JAKi

	**Median (IQR) days to cancer diagnosis**	**n (%) of cancer diagnoses**	**# of cancer diagnoses/ 10,000 person-years (95% CI)**	**Adjusted* HR (95% CI)**
TNFi	238 (154-420)	14 (0.6%)	60 (33-101)	Referent
JAKi	276 (169-351)	22 (0.9%)	100 (63-151)	1.8 (0.9-3.6)

* Adjusted for demographics (age, sex, region, year of initiating biologic), proxies of RA severity (glucocorticoid and csDMARDs prescription fills and number of drug switches), clinical characteristics (days from RA diagnosis to biologic initiation, tobacco use, frailty status, Charlson comorbidity score), and healthcare utilization in the 12 months prior to starting treatment (hospital admissions, emergency department visits, outpatient visits, opioid fills, and NSAID fills).

© 2024 Sendaydiego X et al. *JAMA Network Open.*

eFigure 4. Adjusted Cox Proportional Hazards Curves for Time to Incident Cancer in Those Treated With TNFi Compared to JAKi

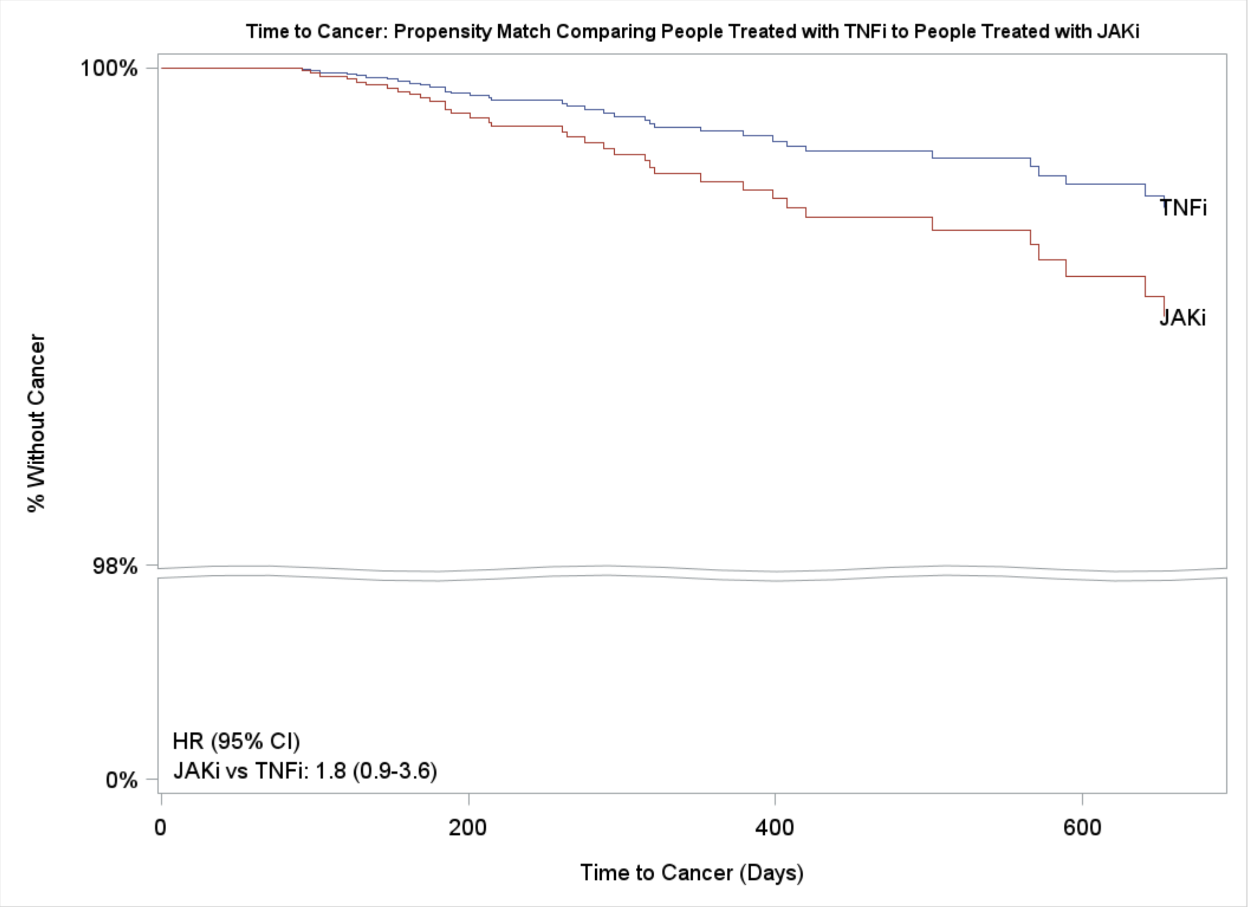

© 2024 Sendaydiego X et al. *JAMA Network Open.*

eTable 11. Baseline Characteristics Comparing Those Who Were Censored to Those Who Were Diagnosed With Cancer

**Variable (No. (%)) unless otherwise specified)**	**Censored (n=27,425)**	**Cancer diagnosis (n=236)**
**Demographics**		
Age in years (median (IQR))	50 (42-55)	53 (48-58)
Male	5,777 (21.1%)	65 (27.5%)
Female	21,648 (78.9%)	171 (72.5%)
Geographic region		
Northeast	3,745 (13.7%)	35 (14.8%)
North Central	5,538 (20.2%)	44 (18.6%)
South	13,596 (49.6%)	119 (50.4%)
West	4,192 (15.3%)	35 (14.8%)
Unknown	354 (1.3%)	3 (1.3%)
Year of treatment exposure (median (IQR))	2016 (2014-2018)	2016 (2014-2018)
**Proxies of RA severity**		
≥1 Fill of glucocorticoids in 3 months prior	12,544 (45.7%)	118 (50.0%)
≥1 Fill of csDMARD in 3 months prior	18,815 (68.6%)	172 (72.9%)
**Clinical characteristics**		
Days from RA diagnosis to treatment exposure (median (IQR))	520 (270-1050)	510 (240-1160)
Tobacco use	2,055 (7.5%)	18 (7.6%)
Frailty score (0 [not at all frail] to 1 [severely frail]) (median (IQR))	0.13 (0.12-0.16)	0.14 (0.12-0.17)
Charlson co-morbidity score		
1-2	23,888 (87.1%)	191 (80.9%)
3-4	2,789 (10.2%)	35 (14.8%)
5+	748 (2.7%)	10 (4.2%)
**Healthcare utilization in 12 months prior**		
≥1 Hospital admission	2,442 (8.9%)	29 (12.3%)
≥1 Emergency department visit	7,423 (27.1%)	63 (26.7%)
≥1 Outpatient visit	27,410 (99.9%)	236 (100%)
≥1 Opioid prescription fill	10,607 (38.7%)	98 (41.5%)
≥1 NSAID prescription fill	14,880 (54.3%)	139 (58.9%)
**Drug Treatment Category**		
TNFi	20,424 (74.5%)	162 (68.6%)
Rituximab	1,168 (4.3%)	14 (5.9%)
IL-6i	1,060 (3.9%)	8 (3.4%)
Abatacept	2,225 (8.1%)	30 (12.7%)
JAKi	2,548 (9.3%)	22 (9.3%)
Days of follow-up (median (IQR))	280 (180-520)	450 (260-700)

© 2024 Sendaydiego X et al. *JAMA Network Open.*
