# Supplementary material for: Use of Biologic or Targeted Synthetic Disease-Modifying Antirheumatic Drugs and Cancer Risk
Source: JAMA Netw Open. 2024 Nov 20;7(11):e2446336. doi: 10.1001/jamanetworkopen.2024.46336 (PMC11579790; doi:10.1001/jamanetworkopen.2024.46336)
Supplement: Supplement 2. — Data Sharing Statement [file jamanetwopen-e2446336-s002.pdf]

Data Sharing Statement

Sendaydiego. Use of Biologic or Targeted Synthetic Disease-Modifying Antirheumatic Drugs and Cancer Risk. *JAMA Netw Open*. Published November 20, 2024.
doi:10.1001/jamanetworkopen.2024.46336

Data
Data available: No
